# Ubiquilin-1 Overexpression Increases the Lifespan and Delays Accumulation of Huntingtin Aggregates in the R6/2 Mouse Model of Huntington's Disease

**DOI:** 10.1371/journal.pone.0087513

**Published:** 2014-01-27

**Authors:** Nathaniel Safren, Amina El Ayadi, Lydia Chang, Chantelle E. Terrillion, Todd D. Gould, Darren F. Boehning, Mervyn J. Monteiro

**Affiliations:** 1 Neuroscience Graduate Program, School of Medicine, University of Maryland, Baltimore, Maryland, United States of America; 2 Center for Biomedical Engineering and Technology, School of Medicine, University of Maryland, Baltimore, Maryland, United States of America; 3 Department of Anatomy and Neurobiology, School of Medicine, University of Maryland, Baltimore, Maryland, United States of America; 4 Department of Neuroscience and Cell Biology, University of Texas Medical Branch, Galveston, Texas, United States of America; 5 Department of Psychiatry, School of Medicine University of Maryland, Baltimore, Maryland, United States of America; 6 Department of Pharmacology, School of Medicine University of Maryland, Baltimore, Maryland, United States of America; Inserm U837, France

## Abstract

Huntington's Disease (HD) is a neurodegenerative disorder that is caused by abnormal expansion of a polyglutamine tract in huntingtin (htt) protein. The expansion leads to increased htt aggregation and toxicity. Factors that aid in the clearance of mutant huntingtin proteins should relieve the toxicity. We previously demonstrated that overexpression of ubiqulin-1, which facilitates protein clearance through the proteasome and autophagy pathways, reduces huntingtin aggregates and toxicity in mammalian cell and invertebrate models of HD. Here we tested whether overexpression of ubiquilin-1 delays or prevents neurodegeneration in R6/2 mice, a well-established model of HD. We generated transgenic mice overexpressing human ubiquilin-1 driven by the neuron-specific Thy1.2 promoter. Immunoblotting and immunohistochemistry revealed robust and widespread overexpression of ubiquilin-1 in the brains of the transgenic mice. Similar analysis of R6/2 animals revealed that ubiquilin is localized in huntingtin aggregates and that ubiquilin levels decrease progressively to 30% during the end-stage of disease. We crossed our ubiquilin-1 transgenic line with R6/2 mice to assess whether restoration of ubiquilin levels would delay HD symptoms and pathology. In the double transgenic progeny, ubiquilin levels were fully restored, and this correlated with a 20% increase in lifespan and a reduction in htt inclusions in the hippocampus and cortex. Furthermore, immunoblots indicated that endoplasmic reticulum stress response that is elevated in the hippocampus of R6/2 animals was attenuated by ubiquilin-1 overexpression. However, ubiquilin-1 overexpression neither altered the load of htt aggregates in the striatum nor improved motor impairments in the mice.

## Introduction

Huntington's disease (HD) is an autosomal dominant, progressive neurodegenerative disorder characterized by chorea, psychiatric disturbances, and cognitive impairment [1], [2]. Despite intensive investigation there is still no treatment to delay or prevent HD.

HD is caused by an expansion of a CAG trinucleotide repeat in exon 1 of the huntingtin (htt) gene [3]. Unaffected individuals have between 14 and 34 CAG repeats in this region, while those afflicted with HD have over 35 repeats [4], [5]. Upon translation, this expansion leads to an aberrantly long tract of polyglutamine (polyQ) residues, which is believed to cause htt protein to misfold and acquire toxic properties [6]. In fact, there appears to be a direct correlation between the length of the CAG expansion and htt protein misfolding/aggregation and toxicity [7], [8]. The accumulation of misfolded huntingtin poses a challenge to cellular proteostasis networks, and impairments in the ubiquitin proteasome system [9]–[11], endoplasmic reticulum associated degradation (ERAD) [12]–[14], and autophagy [15], [16]–[18] has been reported. Efforts to restore these systems are being explored as potential therapy for HD.

Ubiquilins are a conserved family of proteins found in all eukaryotes. Humans and mice each possess four ubiquilin genes, each of which encodes a protein of about 600 amino acids. Ubiquilin proteins function to facilitate protein disposal through the proteasome and lysosomal degradation pathways, the same systems that appear compromised in HD [19]–[24]. Indeed there is growing evidence that links ubiquilin proteins and their genes to a number of neurodegenerative diseases. Ubiquilin proteins are found in the neuropathologic lesions that characterize HD, amyotrophic lateral sclerosis (ALS), Parkinson's disease, and Alzheimer's disease [25]–[28]. Mutations in ubiquilin-2 are linked to an aggressive X-linked form of ALS with dementia [29]. Similarly, mutations in ubiquilin-1 and 4 genes were recently linked to Brown-Vialetto-Van Laere syndrome and ALS, respectively [30], [31]. Overexpression of ubiquilin-1 suppresses polyglutamine toxicity in cell culture and *Caenorhabditis elegans* models of HD, leading to a decrease in htt inclusions and cell death [19]. By contrast, knockdown of ubiquilin expression increases cell death, increases htt aggregates, induces endoplasmic reticulum (ER) stress, impairs autophagosome formation and maturation, and reduces lifespan of flies and nematodes [19], [22], [32], [33].

Here we report on the effects of increasing ubiquilin-1 expression on HD progression in the R6/2 mouse model of HD [34]. We demonstrate that ubiquilin levels decrease progressively and dramatically during late-stages of disease in R6/2 mice. We produced transgenic mice overexpressing human ubiquilin-1 in neurons, which we crossed with R6/2 mice in order to test the hypothesis that restoration of ubiquilin levels would be protective in HD as a potential therapy for HD. We found that ubiquilin-1 overexpression dramatically increased lifespan, delayed formation of htt inclusions and attenuated ER stress in the hippocampus, but it did not improve motor deficits.

## Materials and Methods

### Animal research

This study was carried out in strict accordance with the recommendations in the Guide for the Care and Use of Laboratory Animals of the National Institutes of Health. The protocol was approved by the IACUC committee of the University of Maryland Baltimore.

### Generation of ubiquilin-1 transgenic mice

In order to generate ubiquilin-1 overexpressing transgenic mice an ∼1.8 kb cDNA fragment encoding human ubiquilin-1 fused with FLAG-tag at its N-terminus was inserted into the Thy1.2 expression cassette. The transgenic construct was then linearized with EcoRI and PvuI and used for pronuclear injection into fertilized eggs of the hybrid strain B6C3F2 and inbred strain C57BL/6J. Founder mice were then identified using Southern blotting and polymerase chain reaction (PCR). Two transgenic founder mice, line 48 and 62 were found to carry different copy numbers of the injected transgene. Immunoblots indicated line 62 had higher levels of the ubiquilin-1 overexpression and were backcrossed in a C57BL/6J background for 7 generations.

### Animal husbandry and crosses

Line 62 ubiquilin-1 males were then crossed with ovary-transplanted R6/2 females (strain 006494) carrying 120±5 CAG repeats from Jackson Labs (Bar Harbor, Maine). We used ovary-transplanted females because R6/2 mice are poor breeders. Mice were weaned and tail snipped at postnatal day 21. Following genotyping, mice were regrouped into experimental cages at week 4 containing four mice each, with one of each genotype. The following number of animals for each genotype were used for behavior: WT = 4, UBQ1 = 12, R6/2 = 10, R6/2-UBQ1 = 7. For survival studies, fourteen R6/2 mice were used, of which three were euthanized at 112, 130, and 136 days, and seven R6/2-UBQ1 mice were used, of which three were euthanized at 141, 142, and 164 days as they reached endpoint criteria. The rest were found dead. Four WT and fourteen UBQ1 mice were used for these survival studies, all of which survived the entire duration of the experiment. Data of the behavioral and pathological changes are reported for female animals to avoid potential confounds that can occur when using males [35]. All animals were house in a pathogen free facility at the University of Maryland animal facility and given regular mouse chow and fresh water. Only one person besides the personnel involved in cage maintenance handled the animals for the entire study. The person was blinded to the genotypes. The animals were monitored daily at the beginning of the study and twice daily when animals were close to reaching endpoint criteria. A veterinarian also ensured the health of animals. The criterion for euthanasia was when mice were unable to initiate movement when placed on their side for 20 seconds [36]. No drugs were used for the entire study except for tail biopsies for genotyping purposes. For this procedure the animals were anesthetized by brief inhalation with isoflurane and a small segment of the tail was removed using a razor blade. For euthanasia, mice were placed in an enclosed chamber and exposed to CO_2_ from a regulated tank to effect, followed by cervical dislocation. This same procedure was used for animals used for all biochemical and immunohistochemical analyses.

### Genotyping and CAG Repeat Sequencing

Genomic DNA was extracted from tail tissue using the Puregene Core Kit A (Qiagen, Hilden, Germany). Transgenic progeny were identified by Southern blotting and PCR analysis. For Southern blotting, mouse genomic DNA was digested with EcoRV enzyme and hybridized with ^32^P-labelled with an EcoRV-XhoI fragment, spanning human ubiquilin-1 and 3′ sequences in the Thy1.2 transgenic cassette. The ubiquilin-1 transgene was screened using primers containing sequences specific to the Thy1.2 expression cassette and the ubiquilin-1 cDNA. The transgene was amplified using the sense primer (5′-TCTGAGTGGCAAAGGACCTTA-3′) and the antisense primer (5′-GCTCTAGACTAAGACAAAAGTTGTCGCTGCATCTGACT) at the following cycling conditions: 98°C for 2 min, then 30 cycles (composed of 95°C for 10 sec, 62°C for 15 sec, and 72°C for 90 sec), followed by 72°C for 8 min, and terminating with 4°C. Mice containing the polyglutamine expansion were screened as previously described [35]. The CAG repeat length of R6/2 carriers was determined by Laragen (Los Angeles, CA).

### Body Weight and Survival

Body weight was measured on a weekly basis to the nearest 0.1 g beginning at 8 weeks of age. Survival was measured as reported previously [56].

### Rotarod

Rotarod analysis was performed on a custom built rotarod, set to accelerate from 4 to 40 rpm over a 530-second period. The rod was fitted with a bicycle inner tube to increase traction [35]. Latency to fall was recorded on three trials, with each trial separated by a 30-minute rest period. The trial with the longest latency was scored. At 7 weeks of age mice trained on three consecutive days. Only data from the last day of training was used in statistical analyses. Performance was measured weekly from 7 to 12 weeks of age.

### Grip Strength

Forelimb grip strength was measured weekly from 8 weeks to 13 weeks of age using a Ugo Basile (Varese, Italy) 47106 grip strength meter. Mice were held in front of a grasping bar, which they instinctually gripped onto. Mice were pulled by the tail until their grip strength was overcome, and they lost grip of the bar. Their peak pull force was recorded in each of five trials. The three trials with the highest recorded force were then averaged.

### Open Field

At 10 and 12 weeks of age locomotor activity was assessed using the open field test. To best measure the activity of the mice, trials were conducted one hour after the beginning of their dark cycle. Mice were placed into a 50×50 cm open field box arena, with one mouse from each genotype tested at the same time in different arenas. Total distance traveled was measured over the period of 15 minutes. Between trials, the floor of each chamber was washed with 70% ethanol followed by a ten-minute period where the smell of ethanol was allowed to dissipate. This was done to minimize any odors left by mice on previous trials that could potentially affect exploratory behavior.

### Immunoblotting

Brains were dissected immediately following euthanization. One hemisphere was then homogenized in protein lysis buffer (PLB: 50 mM Tris pH 6.8, 150 mM NaCl, 20 mM EDTA, 1 mM EGTA, 0.5% SDS, 0.5% NP40, sarkosyl 0.5%, 1 mM prefabloc, 10 mM orthovanadate, 2.5 mM sodium fluoride) [37], [38]. Total protein concentrations were then determined using the bicinchoninic acid assay (Thermo Scientific, Rockford, IL). Brain homogenates and lysates were both stored in aliquots at −20°C.

Freshly thawed brain homogenates and cell lysates were each diluted to 15 µg/µL and loaded onto either 8.5 or 10% SDS PAGE gels, transferred to 0.45 µm PVDF membranes (Immobilon-P, Millipore, Billeria, MA) and probed with the following primary antibodies: mouse anti-FLAG (#F3165 Sigma Aldrich, St. Louis, MO), mouse anti-ubiquilin (clone 3D5E2, Invitrogen, Carlsbad, CA) which recognizes ubiquilin proteins, rat anti-BiP (sc-13539 Santa Cruz Biotechnology, Santa Cruz, CA), and goat anti-actin (Santa Cruz Biotechnology), rabbit anti-PDI (#2446 Cell Signaling, Danvers, MA), mouse anti-CHOP (#2895 Cell Signaling). Secondary antibodies used were horse radish peroxidase conjugated goat anti-mouse (#31430), goat anti-rabbit (#31460), goat anti-rat (NA935V GE Healthcare) and rabbit anti-goat (#31492 Thermo Scientific).

### Immunohistochemistry

Immediately following mouse euthanasia one hemisphere of the brain was removed and flash-frozen using dry ice. Hemispheres were stored at −80°C until they were retrieved for sectioning. 25 µm thick coronal sections were cut using a crysostat (Leica Biosystems, Buffalo Grove, IL). Immunohistochemistry was performed as previously described [39]. The following primary antibodies were used at the indicated concentrations: EM48 [40] (1∶100; Millipore, Billerica, MA), mouse anti-ubiquilin-1 (1∶1000; Invitrogen, Carlsbad, CA). Alexafluor (Invitrogen) secondary antibodies were used at the following dilutions donkey-anti-mouse 488 (1∶500), goat anti-mouse 594 (1∶500), donkey anti-rabbit 488 (1∶500). In order to quantify inclusion bodies, 6 sections containing the striatum and 6 sections containing the hippocampus per animal were analyzed under an inverted Leica DMIRB fluorescent microscope using a 40× objective. Images were captured using a Hamamatsu digital C8484 camera using iVision software (BioVision Technologies, Exton, PA). In order to reduce bias, a script, which counted the number of puncta above a certain intensity threshold and then filtered them according to size, was ran in the program iVision (BioVision). Number of inclusions was then averaged.

Colocalization of ubiquilin with htt inclusions was carried out using a Zeiss 510 confocal microscope using 405 (DAPI) 488, and 594 nm laser lines.

### Statistical Analyses

Students T-Test and Repeated Measures ANOVA were performed using Microsoft Excel and GraphPad Prism.

## Results

### Ubiquilin levels decline dramatically during late-stages of HD

**Figure 1:**
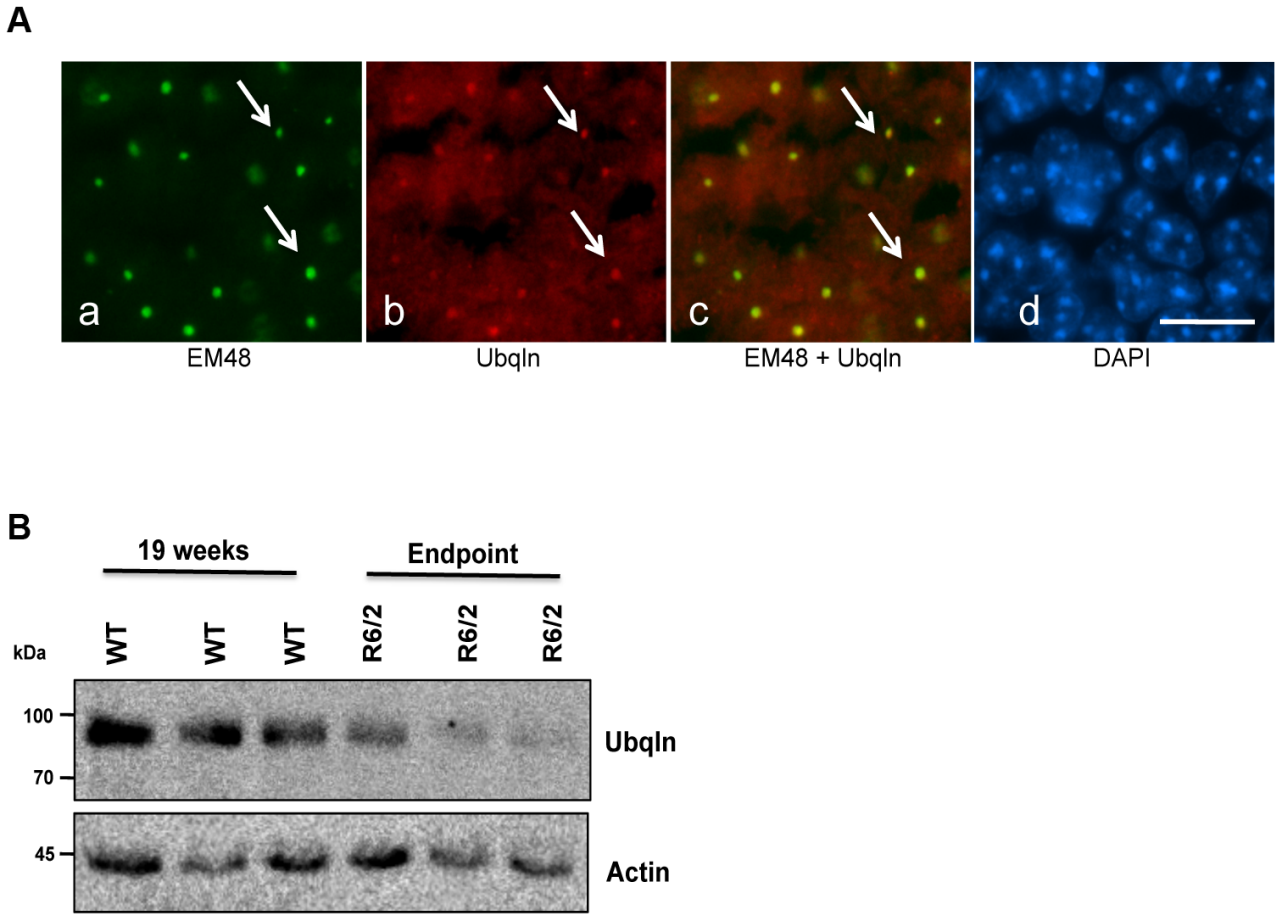
Ubiquilin is found in htt inclusions and its levels decrease in the R6/2 model of HD. (A) Confocal staining of a 15-week old end-stage R6/2 brain section of the hippocampus showing staining with anti-ubiquilin (red), anti-htt EM48 (green) and DAPI (blue). The merged image of the red and green fluorescence images shows ubiquilin colocalizes with htt inclusions (arrows). Bar = 15 µm. (B) Equal amounts of protein in whole brain homogenates from three end-stage R6/2 mice and three 19 week-old WT mice were immunoblotted for ubiquilin and for actin. Note the decline in ubiquilin levels in end-stage R6/2 animals.

**Figure 3:**
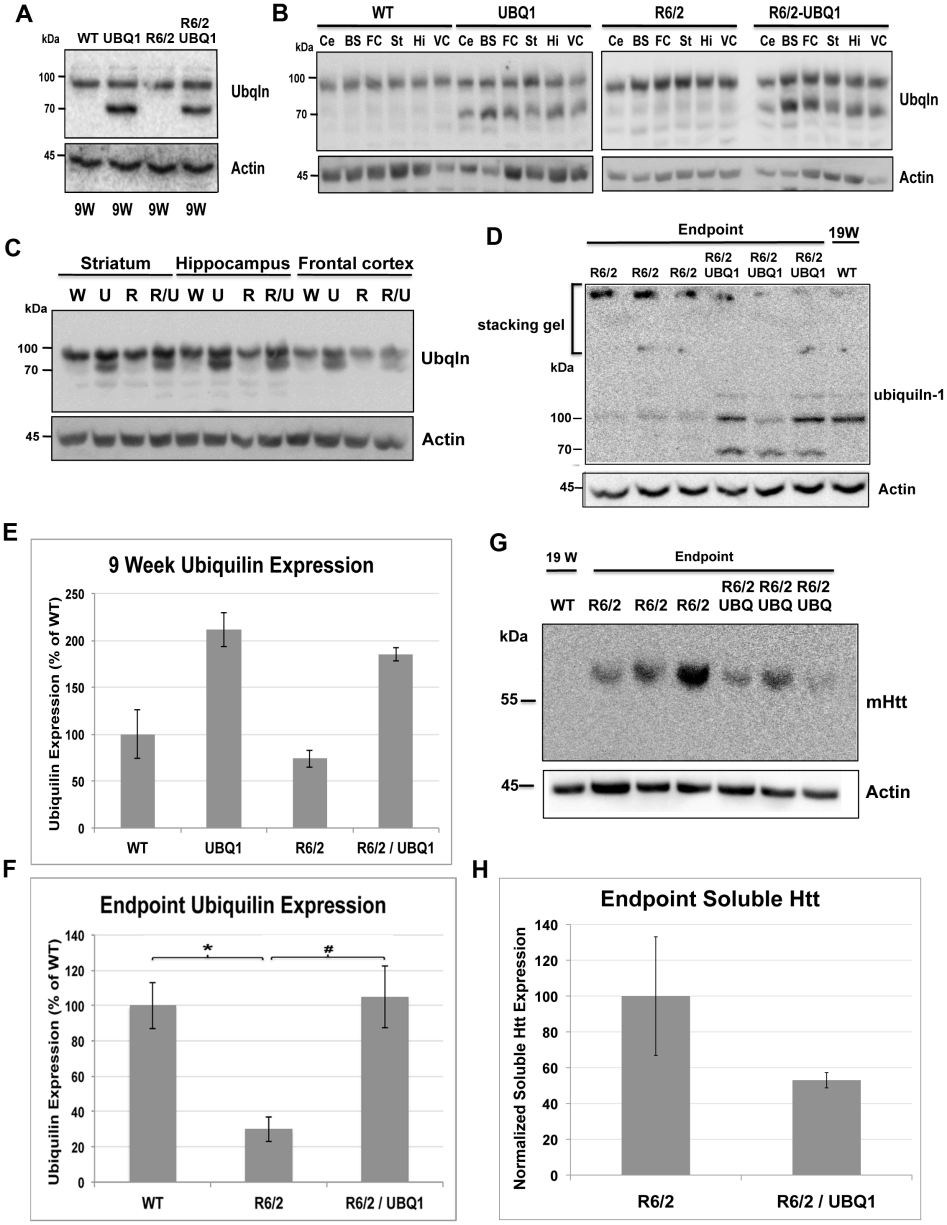
Restoration of ubiquilin levels in animals carrying the R6/2 transgene and accompanying changes in htt protein levels. (A) Immunoblots of lysates made from dissected brain regions of 9 week-old mice with different genotypes obtained after crossing UBQ1 transgenic mice with R6/2 mice and probed for ubiquilin (upper panel) and for actin (lower panel). Note successful overexpression of ubiquilin in animals carrying the ubiquilin-1 transgene. (B) Immunoblot analysis similar to A, but this time showing ubiquilin levels in different regions of the brain (Ce  =  cerebellum, BS  =  Brain stem, FC  =  frontal cortex, St  =  striatum, Hi  =  hippocampus, VC =  visual cortex). Note overexpression of ubiquilin in all tissues of transgenic mice carrying the ubiquilin-1 transgene. (C) Immunoblots of equal amounts of protein in lysates made from the striatum (St), hippocampus (Hi) and frontal cortex (FC) of 9 week-old mice from the same cross mentioned above (W = non-transgenic for UBQ1 or R6/6, U = UBQ1 transgenic, R = R6/2 transgenic, R/U = R6/2-UBQ1 double transgenic) and probed for ubiquilin or actin. (D) Immunoblots of brain lysates from end-stage mice showing R6/2-UBQ1 double transgenic mice have higher ubiquilin levels detected in the resolving gel than R6/2 animals, which correlated with decreased ubiquilin that was trapped in aggregates in the stacking gel. Also included is a lysate from a 19-week old WT animal (right lane). (E and F) Quantification of ubiquilin levels in 9-week (E) and end-stage or equivalent time-point animals (F) showing the amount of ubiquilin protein expression in animals with different genotypes used in our study. Note that ubiquilin levels are reduced by 70% in end-stage R6/2 animals compared to wild type age-matched controls (*p* = 0.0011). Furthermore, ubiquilin levels were fully restored in the brains of end-stage R6/2-UBQ1 double transgenic mice compared to R6/2 transgenic mice (*p* = 0.0017). (G) Immunoblot of same lysates shown in D with EM48 antibody showing ubiquilin overexpression in R6/2-UBQ1 double transgenic mice have reduced levels of soluble mutant htt protein accumulation compared to R6/2 animals. Note the WT lysate was loaded on the left lane. (H) Quantification of soluble htt protein shown in panel G.

The R6/2 HD mouse model is used extensively in HD therapeutic trials due to its relatively short life span and ability to recapitulate many of the symptoms and underlying pathology of HD [35]. R6/2 mice express exon 1 of the human HD gene containing approximately 120 CAG repeats [34]. Inclusion bodies can be detected in R6/2 mouse brains as early as 3 weeks of age, which is followed by a rapid decline in behavior, motor and cognitive function, and culminating in premature death at approximately 4 months of age [34], [41], [42]. Previously we demonstrated that knockdown of ubiquilin-1 expression in Htt-Q74 expressing stable cell lines increases cell death [19]. Moreover, knockdown of ubiquilin expression slows proteasome degradation [21], [22] and disrupts autophagy [23], [24], the same two pathways that appear to be compromised in HD. We first examined whether ubiquilin proteins are contained in huntingtin aggregates by immunohistochemistry. Similar to previous findings in mouse and human brain [27], [43], [44], we found ubiquilin staining was colocalized with htt inclusions in brain sections of end-stage R6/2 animals (Figure 1A). We next examined whether ubiquilin levels change during disease progression in R6/2 mice. Immunoblotting of whole brain lysates with a monoclonal specific ubiquilin antibody revealed a dramatic 70% decrease in ubiquilin levels at end stage of disease (Figure 1B and 3F). The results suggest a negative correlation between ubiquilin protein accumulation and HD.

### Generation and characterization of ubiquilin-1 transgenic mice

**Figure 2:**
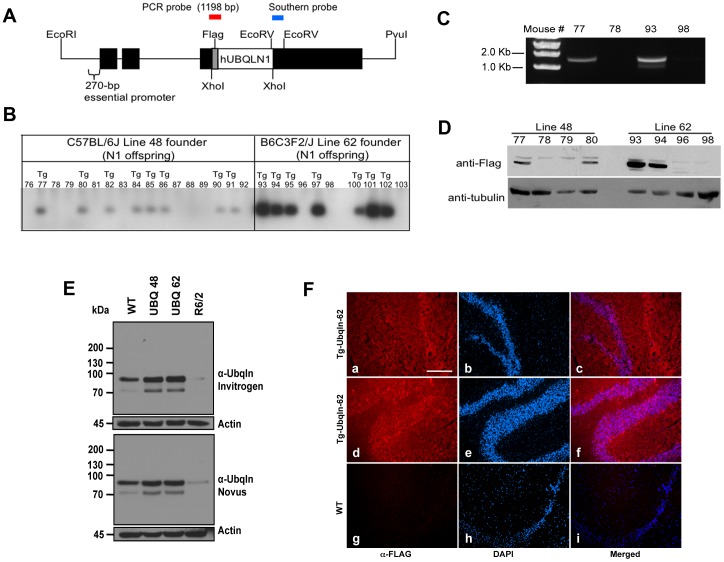
Generation of transgenic mice that overexpress human ubiquilin-1. (A) Schematic of the Thy1.2 expression construct used to generate ubiquilin-1 transgenic mice. Human ubiquilin-1 with an N-terminal FLAG tag was cloned in the appropriate orientation between the XhoI site of the Thy1.2 expression cassette. (B) Southern Blot of the first generation offspring of two founder mice (48 and 62). (C) Validation of a PCR genotyping protocol. Amplification of the transgene was only observed in mice that Southern blotting revealed to be positive. (D) Immunoblots of brain cortical lysates with an anti-FLAG antibody and for tubulin indicated that line 62 offspring express higher levels of FLAG-ubiquilin-1 than line 48. (E) Immunoblots of equal amounts of total brain lysates from 12 month-old WT mouse, 12 month-old Ubqln-1 48 transgenic mouse, 12 month-old Ubqln-1 62 transgenic mouse and end stage 15 week-old R6/2 transgenic mouse. The top panel was probed with a monoclonal anti-ubiquilin antibody (Invitrogen antibody clone 3D5E2) and the lower panel with a different monoclonal anti-ubiquilin antibody (Novus antibody clone 5F5). Note two immunoreactive ubiquilin bands are seen at ∼70 kDa and at ∼90 kDa, which we presume is a modified form of ubiquilin. Both blots were also probed for actin to ensure equal loading. (F) Cryostat sections of a Ubqln-1 62 transgenic mouse brain (a–f) and WT mouse brain (g-i) showing anti-FLAG antibody staining (Alexa 594, left panels) and corresponding DAPI staining (center panels) and the result of merging the fluorescent and DAPI signals (right hand panels). The brain sections shown are of the hippocampus (a–c and g–i) and cerebellum (d–f). Identical exposure settings were used for the left hand panels.

In order to test whether increasing ubiquilin expression would be protective in HD, we generated transgenic mice that express N-terminal FLAG-tagged human ubiquilin-1 under the control of the Thy1.2 promoter (Figure 2A), which drives transgene expression specifically in neurons [45]–[47]. Founders were identified via Southern blotting (Figure 2B) and subsequently confirmed via diagnostic PCR analysis (Figure 2C). Two transgenic lines were further characterized, line 62 and line 48 (Figure 2D). Immunoblots of brain lysates indicated that total ubiquilin levels in line 62 were increased approximately 200% compared to control nontransgenic mice (Figure 2E and 3E), whereas ubiquilin levels were lower in line 48 when the different bands seen in the blots were considered together (Figure 2E). Both lines expressed appropriate size FLAG-tagged human ubiquilin-1 protein that migrated on gels with a mass of ∼68 kDa, similar to what we had shown previously [25]. Interestingly, the monoclonal specific ubiquilin antibody reacted with multiple bands in the mouse brain lysates, including the ∼68 kDa band and a more prominent band at ∼90 kDa, which we presume reflects some post-translation modification of the proteins (Figure 1E and 3A). Further immunoblots of different brain regions, as well as immunohistochemistry revealed global overexpression of the ubiquilin-1 transgene in the brain (Figure 2E, F and 3B and C). All subsequent studies were carried out using the higher expressing line 62 mice (henceforth referred to UBQ1 mice).

More detailed characterization of the UBQ1 62 transgenic mice (as well as the 48 line) indicated they were completely normal in terms of appearance, lifespan and according to a battery of behavioral tests (rotarod, grip strength and open field analysis). The mice therefore possessed the appropriate characteristics, overexpression of ubiquilin-1 with no unintended detrimental effects, making them suitable for crossing with mouse models of HD.

### Restoration of ubiquilin levels in R6/2 mice brains by transgenic overexpression of ubiquilin-1

Because the R6/2 model is widely used to test therapeutic candidates for HD we crossed our UBQ1 62 transgenic line with R6/2 mice and obtained progeny with appropriate Mendelian transmission of the two transgenes. Comparison of ubiquilin levels in various brain regions of 9-week old animals in each of the four resulting genotypes revealed higher expression in the animals that inherited the ubiquilin-1 transgene, as expected (Figure 3B and C). Importantly, the increase was seen in R6/2-UBQ1 double transgenic mice, indicating successful overexpression of ubiquilin-1 in the presence of the R6/2 transgene (Figure 3A–C). Moreover, immunoblots of brain lysates from end-stage HD-affected R6/2-UBQ1 double transgenic mice revealed ubiquilin levels had been fully restored to the amount that is typically found in age matched wild type animals (Figure 3D and F). An immunoblot with EM48 antibody, which selectively recognizes human mutant huntingtin protein, revealed an ∼50% decrease in mutant soluble huntingtin protein in R6/2-UBQ1 double transgenic mice compared to R6/2 animals (Figure 3G and H). However, the changes were not statistically significant at this time-point. There was variability in ubiquilin expression in the mice that inversely correlated with huntingtin protein accumulation. The consequence of increased ubiquilin-1 expression was further evaluated by conducting a battery of tests on the mice, including monitoring effects on survival, body weight, behavioral phenotypes and neuropathology. Changes in all of these parameters are strongly linked with the transmission of the R6/2 transgene [35], [48].

### Ubiquilin-1 overexpression increased survival of R6/2 mice, but had no effect on motor behavior or body weight

The length of polyglutamine tracts in R6/2 mice has been shown to have a profound effect on phenotype [49]. Moreover, polyglutamine expansions are unstable and have the potential to increase in size upon transmission to progeny [35], [50]. In order to ensure that all experimental mice had comparable numbers of repeats the polyglutamine region was sequenced in all R6/2 carriers. The measurements indicated that R6/2 single and R6/2-UBQ1 double transgenic mice had, on average, the same number of CAG repeats (R6/2 (n = 18): Mean = 125, SD = 2, R6/2-UBQ1 (n = 10): Mean = 125, SD = 2).

**Figure 4:**
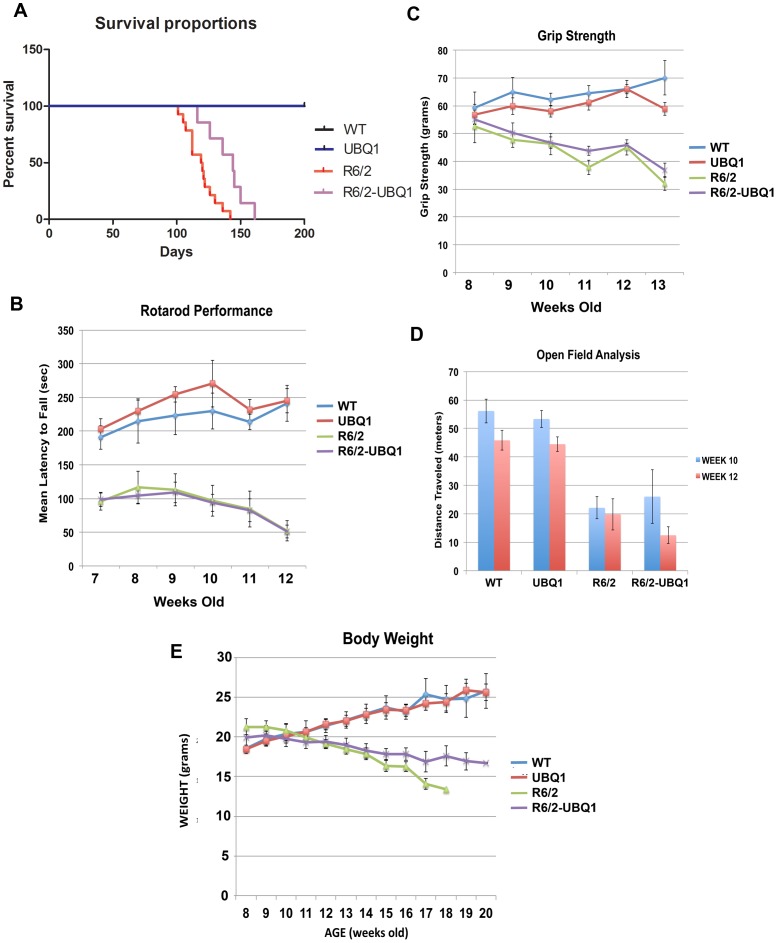
Ubiquilin-1 Overexpression increased survival but had no effect on motor function or loss of body weight. (A) Kaplan-Meier survival curve showing a 20% increase in lifespan in double transgenic mice (R6/2: N = 14, R6/2-UBQ1: N = 7, *p* = 0.0032). (B–D) Ubiquilin-1 overexpression failed to improve rotarod performance (B), grip strength (C) and activity in the open field (D), at any time point tested. (E) There was no significant effect of ubiquilin-1 overexpression on weight loss. For all behavioral experiments: (WT: N = 4, UBQ1: N = 12, R6/2: N = 10, R6/2-UBQ1: N = 7).

To eliminate other possible confounds, all of the animals were housed, handled and tested using the recommended guidelines for R6/2 mice [35]. Because of the aggressive nature of male mice, which can profoundly affect the behavior and well being of cage mates, the results presented are those for females only, although similar effects were obtained when the data from both sexes were combined. Ubiquilin-1 overexpression significantly improved survival of R6/2 mice (Figure 4A). Mean survival of R6/2-UBQ1 mice increased by 20% compared to R6/2 mice (R6/2: 119.5 days, n = 14, SEM = 3.16, R6/2-UBQ1: 144 days, n = 7, SEM = 5.71; *p* = 0.0032). None of mice lacking the R6/2 transgene died before the longest surviving R6/2 carrier.

Despite this increase in lifespan, R6/2-UBQ1 mice did not exhibit improved motor function. The rotarod test was performed on a weekly basis in order to assess balance and coordination (Figure 4B). Repeated Measures ANOVA revealed a main effect of genotype (*F*_3, 108_ = 22.68, *p*<0.0001). Bonferroni posttests found significant differences between WT and R6/2 at weeks 7 and from 9 to 12, and between WT and R6/2-UBQ1 mice at all time points. Statistical analysis of the data indicated no significant difference between R6/2 and R6/2-UBQ1 mice at any time point. Therefore, we conclude that ubiquilin-1 overexpression had no effect on rotarod performance.

We observed a similar trend when measuring grip strength (Figure 4C). Repeated Measures ANOVA showed a main effect of age (*F*_5, 150_ = 2.48, *p* = 0.0341), genotype (*F*_3, 150_ = 32.92, *p*<0.0001), as well as an interaction between age and genotype (*F*_15, 150_ = 2.94, *p* = 0.0004). Bonferroni posttests revealed significant differences between WT and R6/2 from weeks 9 to 13, and between WT and R6/2-UBQ1 mice from weeks 10 to 13. However, no significant differences were observed between R6/2 and R6/2-UBQ1 mice at any time point.

R6/2 mice initially display hyperactivity at 3 weeks of age and later exhibit hypoactivity beginning at 7 weeks, which becomes more pronounced with age [51]. In order to determine whether ubiquilin-1 overexpression had an effect on locomotor activity the open field test was performed initially at 10 weeks, and then repeated at 12 weeks of age (Figure 4D). A main effect of testing session (*F*_1, 23_ = 27.71, *p*<0.0001), and genotype (*p*<0.0001), was observed. However, post-hoc tests failed to reveal a difference between R6/2 and R6/2-UBQ1 mice at either time point, indicating that ubiquilin-1 overexpression has no significant effect on locomotor activity. Finally, ubiquilin-1 overexpression also failed to significantly improve body weight at any time point (Figure 4E).

### Ubiquilin-1 overexpression delays huntingtin aggregates in the hippocampus, but not the striatum

**Figure 5:**
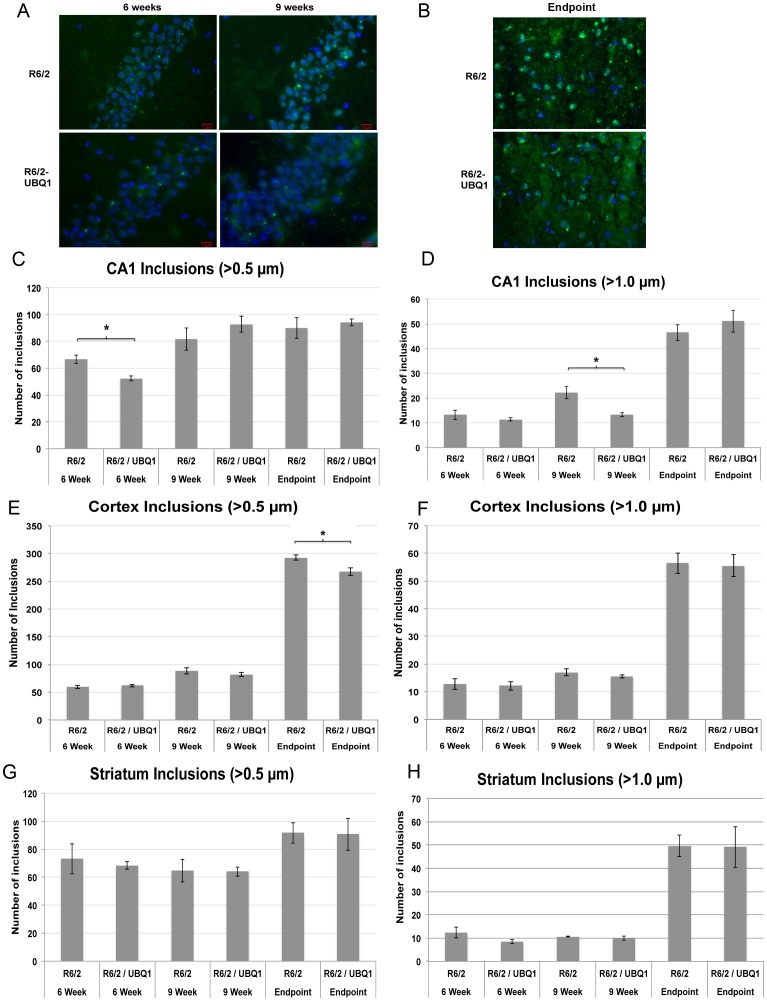
Ubiquilin-1 overexpression modifies aggregate load in the hippocampus and cortex but not the striatum. (A) Representative fluorescence microscopy images of EM48 and DAPI stained cryostat sections of the CA1 region of the hippocampus in R6/2 transgenic and R6/2-UBQ1 double transgenic mouse at 6 weeks, 9 weeks and following end-stage euthanasia. Bar = 15 µm. (B) Similar to A, but showing representative sections from the dentate gyrus in end-stage mice. Bar = 15 µm. (C) Quantification of htt inclusions >0.5 µm in size in the CA1 region of the hippocampus at 6 weeks, 9 weeks and end-stage R6/2 and R6/2-UBQ1 double transgenic mice. The R6/2-UBQ1 double transgenic mice contained 22% fewer inclusions than R6/2 mice at 6 weeks (*p = *0.04), but not at the other times. (D) Quantification of htt inclusions >1 µm in size in the CA1 region of the hippocampus at 6 weeks, 9 weeks and end-stage R6/2 and R6/2-UBQ1 double transgenic mice. The R6/2-UBQ1 double transgenic mice had 40% fewer inclusions at 9 weeks compared to R6/2 transgenic mice (*p* = 0.027). (E and F) Similar to B and C, but showing htt inclusions in the cortex. R6/2-UBQ1 double transgenic mice had 8.5% fewer inclusions greater than 0.5 µm at the end-stage of disease. (G, H) Similar to E and F, but comparing inclusions in the striatum. There was no difference in the number of inclusions in the striatum between the two genotypes at any time point.

Previously we demonstrated that ubiquilin-1 overexpression suppresses htt aggregation in cell culture [19]. We therefore explored whether ubiquilin-1 overexpression could reduce or delay the formation of inclusion bodies in the brains of R6/2 mice. We tested several methods to quantify the changes in huntingtin protein aggregation. We found immunohistochemistry was the most reliable and reproducible method to quantify aggregates. iVision software was used to identify inclusions that were greater than 0.5 µm in diameter. We then filtered the inclusions to identify those greater than 1.0 µm in diameter, enabling us to quantify small inclusions (<1.0 µm) from large inclusions (>1.0 µm). We observed a 22% reduction in the number of total inclusions in the CA1 region of the hippocampus of 6 week-old R6/2-UBQ1 mice (N = 3, *p* = 0.04) (Figure 5A and C). This was primarily due to 24% reduction of small inclusions. This was followed by a 40% reduction in large inclusions in CA1 at 9 weeks of age (N = 3, *p* = 0.027) (Figure 5A and D). Presumably, the reduction in large inclusions at week 9 was due to a smaller pool of inclusions with which to expand at week 6. In the cortex we observed a similar trend during this period and a statistically significant reduction of 0.5 µm diameter inclusions in end-stage animals (Figure 5B, F and G). Ubiquilin-1 overexpression failed to modify htt aggregation at any time point in the striatum (Figure 5G and H).

### Overexpression of ubiquilin-1 attenuates ER stress in the hippocampus

**Figure 6:**
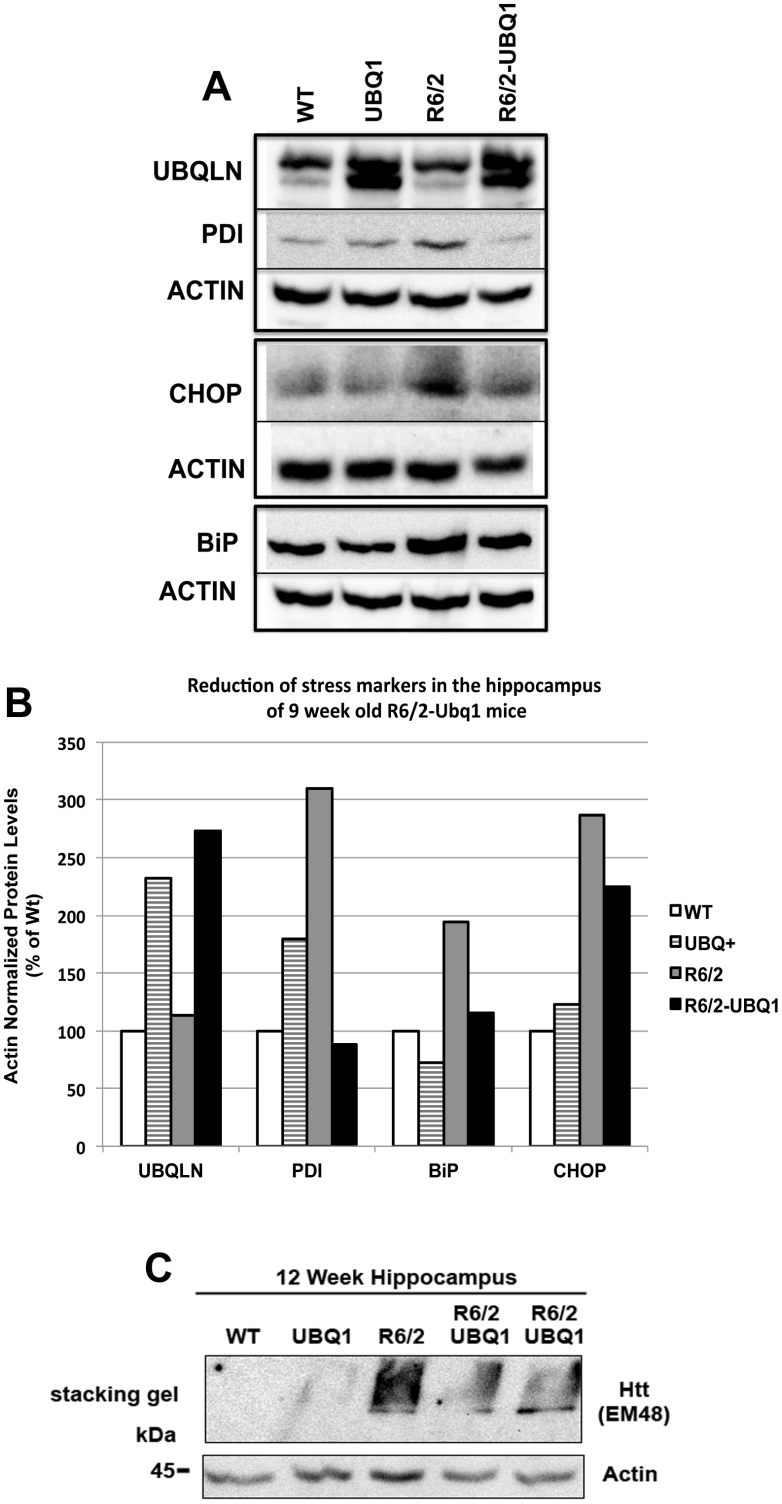
Ubiquilin-1 overexpression attenuates ER stress in R6/2 animals. (A) Immunoblots of equal amounts of brain lysates from 9 week-old female animals from the R6/2 and UBQ1 cross. The blots were probed for PDI, BiP, ubiquilin, and CHOP as well as for actin to monitor protein loading. (B) Quantification of the expression of the different proteins in the four mice after normalization for actin loading. (C) Immunoblot of 12-week hippocampal lysates to detect htt aggregates retained in the stacking gel. The double transgenic mice had an approximately 30% reduction in EM48 immunoreactivity after normalization for actin loading.

Because ubiquilin-1 facilitates ERAD [21], [22] and because htt proteins containing polyglutamine expansions have been implicated in disruption of ERAD [12], [14] we next investigated whether mice overexpressing ubiquilin-1 had altered ER homeostasis. Interference of ERAD can trigger the unfolded protein response (UPR), which involves the coordinated activation of a series of signaling pathways that function to restore ER homeostasis [52]. Several proteins are activated during UPR, so measuring fluctuations in their levels is used to monitor ER stress [53]–[55]. We focused on three classical ER stress markers: BiP (immunoglobulin-binding protein or grp78), PDI (protein disulfide isomerase) and CHOP (transcription factor C/EBP homologous protein) [56]. BiP and PDI are two ER chaperones that are activated during UPR to restore protein folding in the ER, whereas activation of CHOP signals execution of the cell death or apoptosis program [55]. Accordingly, we examined if these three proteins were altered by ubiquilin-1 overexpression in the hippocampus of 9 week-old animals. We focused on the hippocampus because it is where we found distinct changes in htt aggregation from ubiquilin overexpression. The immunoblots indicated that BiP, PDI and CHOP were all elevated in R6/2 mice, compared to non-transgenic or ubiquilin-overexpressing mice (Figure 6A and B). More interestingly overexpression of ubiquilin-1 attenuated the increase in each of these stress markers in ubiquilin-1-overexpressing R6/2 mice (Figure 6A and B). Further analysis of hippocampal lysates for huntingtin protein trapped in the stacking gel confirmed EM48 immunoreactivity was reduced in R6/2-UBQ1 mice compared to an R6/2 animal (Figure 6C).

## Discussion

Our study provides direct in vivo evidence that increasing ubiquilin-1 expression may be beneficial for HD. The most compelling evidence is that through transgenic overexpression of human ubiquilin-1 protein, mean lifespan of R6/2 mice was increased by 20%. The observed increase in lifespan is amongst the largest improvements in survival in R6/2 therapeutic trials [57]. In fact, most of the large improvements in R6/2 survival have come from drug treatment of animals, and not by transgenic manipulation of proteins, as we have done here. Besides survival, ubiquilin-1 overexpression delayed inclusion body formation in the CA1 region of the hippocampus and cortex. Finally, consistent with a functional role of ubiquilin-1 in promoting ERAD, R6/2-UBQ1 double transgenic mice overexpressing ubiquilin-1 had an attenuated ER stress response in the hippocampus compared to an age-matched mouse carrying the R6/2 transgene alone. There are several possible explanations for our findings.

Because the exact mechanisms by which huntingtin proteins containing polyglutamine expansions cause disease is still not known, it is difficult to know if the increase in R6/2 survival from ubiquilin-1 overexpression is related to any specific improvement in a specific pathway(s). However, it is clear that it must represent some improvement in a crucial pathway(s) needed for survival. Two hints suggested by our study is the possible relationship of survival to formation of htt inclusions and/or to an attenuation of ER stress. The reduction in htt aggregates found in the hippocampus and cortex is consistent with our previous findings conducted in cell culture and *C. elegans* showing ubiquilin overexpression reduces htt aggregates and improves a motility defect in nematodes [19]. Moreover, previous studies have shown ubiquilin-1 overexpression increases the turnover of expanded huntingtin protein [20]. Thus a simple explanation for our findings is that R6/2-UBQ1 mice overexpressing ubiquilin-1 have increased turnover of the polyQ-expanded htt protein thereby reducing the amount of mutant htt protein available for aggregation and for inducing toxicity. Consistent with this idea we found ubiquilin-1 overexpression led to a reduction in accumulation of soluble mutant htt protein in the mice (Figure 3E).

A conundrum was why huntingtin inclusions in the striatum were not reduced despite clear evidence of ubiquilin overexpression? Although we do not know the answer we speculate that intrinsic differences in the composition and/or function of neurons in the CA1 region of the hippocampus and cortex compared to the striatum could influence the ability of htt protein to aggregate in one cell type, but not the other. It is interesting that the reduction in inclusions we found occur in regions of the brain that are rich in pyramidal neurons, which suggest that they could be more amenable to rescue by ubiquilin-1 overexpression.

The attenuation of ER stress in R6/2 mice overexpressing ubiquilin-1 is most likely related to facilitation of ERAD by ubiquilin [21], [22]. For example, studies have shown that overexpression of ubiquilin-1 enhances degradation of ERAD substrates, whereas knockdown of ubiquilin-1 expression slows degradation [21], [22]. In accordance with facilitating ERAD, knockdown of ubiquilin in *C. elegans* induces ER stress [22]. Thus, our results are consistent with ubiquilin-1 overexpression attenuating ER stress. Activation of ER stress is associated with induction of the unfolded protein response to restore ER homeostasis, but if unsuccessful, its persistent activation can trigger cell death [53], [54], [58], [59]. In fact, there is growing evidence that ER stress could be involved in the etiology of many human diseases, particularly neurodegenerative diseases, such as Huntington's disease [52], [60]. For example, htt proteins with expanded polyglutamine tracts have been shown to interfere with ERAD resulting in induction of ER stress [12], [14]. Furthermore, ER stress markers are increased in different mouse models of HD [61], including the R6/2 model we have used [62]. It remains to be determined whether the increased survival of R6/2-UBQ1 animals is related to alleviation of ER stress and/or whether it is related to the protective effects of ubiquilin in some other pathways.

Despite reducing htt aggregation, attenuating ER stress and increasing mouse survival, we found mice overexpressing ubiquilin-1 had no significant improvement in the deterioration of motor function caused by the R6/2 transgene. Several reasons may account for this apparent discrepancy. The R6/2 mutant huntingtin transgene is ubiquitously expressed [34], [63], while expression of our ubiquilin-1 transgene is restricted to neurons. It is possible that degeneration of muscle tissue (or other cell types) may have occluded any potential improvements in neurological function. Another possibility is that the R6/2 model, which expresses high levels of the toxic exon-1 fragment of htt, might have too aggressive and penetrant phenotypes that could mask subtle improvements from ubiquilin-1 overexpression. Mice with more modest behavioral and pathological phenotypes have been generated by expressing full-length htt containing polyQ expansions at more physiological levels, such as the BACHD and YAC128 transgenic models or the knock-in CAG140 and CAG150 models [64]–[67]. It is therefore possible that ubiquilin-1 overexpression would show some benefit when tested in these less aggressive mouse models of HD. On the other hand, the behavioral impairments in R6/2 mice might be reduced or eliminated by even higher overexpression of ubiquilin-1, either by generating new ubiquilin-1 transgenic mice or by crossing our mice to obtain homozygous transmission of the ubiquilin-1 transgene.

The ubiquilin transgenic mice we generated overexpress approximately 2-fold more ubiquilin protein in total brain lysates. Because the brain lysates are composed of neuronal and non-neuronal cells, and because expression of the Thy1.2 promoter is restricted to neurons, it is likely that ubiquilin-1 overexpression in neurons is even higher. Despite this large increase we have not noticed any overt toxic effects of its overexpression, suggesting any effort to increase ubiquilin overexpression for therapeutic treatment of neurodegenerative disease would likely be safe. Obviously, overexpression in cells other than neurons would have to be tested.

An interesting observation regarding ubiquilin proteins in brain is the presence of a major protein band of approximately 90 kDa that is seen in addition to the normal ∼68 kDa band. The ∼90 kDa band most likely reflects some post-translation modification of ubiquilin. It will be interesting to determine the nature of this modification, and whether ubiquilin function(s) are altered by the presence or absence of the modification.

In summary, our studies suggest that overexpression of ubiquilin-1 provides some benefit when tested using the aggressive R6/2 model of HD. Similar tests conducted with other less aggressive HD mouse models should reveal whether methods to increase ubiquilin expression or activity would be effective for HD treatment.
